# Identification of Novel Markers of Mouse Fetal Ovary Development

**DOI:** 10.1371/journal.pone.0041683

**Published:** 2012-07-26

**Authors:** Huijun Chen, James S. Palmer, Rathi D. Thiagarajan, Marcel E. Dinger, Emmanuelle Lesieur, Hansheng Chiu, Alexandra Schulz, Cassy Spiller, Sean M. Grimmond, Melissa H. Little, Peter Koopman, Dagmar Wilhelm

**Affiliations:** Division of Molecular Genetics and Development, Institute for Molecular Biosciences, The University of Queensland, Brisbane, Australia; Temasek Life Sciences Laboratory, Singapore

## Abstract

In contrast to the developing testis, molecular pathways driving fetal ovarian development have been difficult to characterise. To date no single master regulator of ovarian development has been identified that would be considered the female equivalent of *Sry*. Using a genomic approach we identified a number of novel protein-coding as well as non-coding genes that were detectable at higher levels in the ovary compared to testis during early mouse gonad development. We were able to cluster these ovarian genes into different temporal expression categories. Of note, *Lrrc34* and *AK015184* were detected in XX but not XY germ cells before the onset of sex-specific germ cell differentiation marked by entry into meiosis in an ovary and mitotic arrest in a testis. We also defined distinct spatial expression domains of somatic cell genes in the developing ovary. Our data expands the set of markers of early mouse ovary differentiation and identifies a classification of early ovarian genes, thus providing additional avenues with which to dissect this process.

## Introduction

Despite the importance of the ovary for reproduction through the production of oocytes and the secretion of female sex hormones, its development during embryogenesis remains poorly understood. Ovaries and testes develop from a common origin, the paired genital ridges, that arise at the ventro-medial surface of the mesonephroi at around 10 days *post coitum* (dpc) in mice. Shortly after, the expression of the male-determining gene *Sry* (sex-determining region of chromosome Y) is initiated in the XY gonad, driving the differentiation of the genital ridges into testes [1], [2]. *Sry* expression induces the differentiation of Sertoli cells, which are considered to be the organizers of testis differentiation. They produce key signalling molecules that influence the differentiation of other testicular cell types leading to characteristic testicular histology [3]. If *Sry* is absent or fails to act in time, the indifferent gonad differentiates into an ovary, which is driven by a different gene expression program.

Genomic approaches have identified a number of genes showing XX-specific expression in the developing gonads [4], [5]. For a handful of these genes, functions during early ovary differentiation have been elucidated by generating null mutations in mice [6]–[11]. Despite the search for a master regulator that is both necessary and sufficient for ovarian development, no *Sry*-equivalent has been identified to date. In mouse, null mutations of *Wnt4* (wingless-related MMTV integration site 4), *Rspo1* (R-spondin homolog 1) and *Foxl2* (forkhead box L2), resulted in partial XX sex reversal, suggesting that more than one pathway is required for ovary determination. However, in other species, such as *Foxl2* in goat [12] and *RSPO1* in humans [13], mutations in these genes can result in complete ovary-to-testis sex reversal.

During mouse embryonic development, testes undergo massive morphological changes, whereas ovaries remain relatively small and histologically undifferentiated. The first histologically observable rearrangement occurs around 12.5–13.5 dpc when primordial germ cells (PGCs) form germ cell cysts or nests [14]. At around the same time PGCs start to enter meiosis in an anterior-to-posterior wave [15], [16], whereas in the testis PGCs go into mitotic arrest [17]. Only after birth will the germ cell cysts in an ovary break apart. The majority of the germ cells will then be removed by apoptosis, while remaining individual germ cells recruit somatic cells to give rise to primordial follicles [18]. While the molecular pathways driving early mouse ovarian development are not well characterised, the mechanisms regulating follicular growth in mammalian ovaries during late gestation and postnatally have been elucidated in great detail (for review see [19]).

The soma of the mature, adult ovary consists of granulosa cells, the supporting cell lineage that surround and nurture the developing oocytes, as well as steroidogenic theca cells, stromal and endothelial cells. In the embryonic mouse ovary, only PGCs can be identified easily based on their round morphology. It has not been possible to distinguish between different somatic cell types. The precursors of granulosa cells have been visualised in the early ovary through the use of a transgenic mouse expressing green fluorescence protein under the control of the *Sry* regulatory region [20], however no endogenous marker gene for pre-granulosa cells had been identified. Recently, it has been shown that FOXL2-positive cells in the embryonic ovary give rise to granulosa cells of follicles in the medulla that begin to grow immediately after birth, whereas granulosa cells of the cortical follicles seem to arise from proliferating cells in the surface epithelium [21]. Under the influence of factors secreted by the developing oocytes, these pre-granulosa cells differentiate into mature granulosa cells [22]. The origin of the theca cells is even less clear. They are first observed after birth when a follicle consists of two or more layers of granulosa cells [23]. It is assumed that signals from the growing follicles stimulate the differentiation of theca cells from progenitor populations [24] and subsequently induce the expression of the luteinizing hormone receptor. In response to luteinizing hormone, theca cells produce androgens, which are then converted into estrogen by granulosa cells. Similar to pre-granulosa cells, there is no marker gene known that is specific for theca progenitor cell populations. Taken together, it is not possible to distinguish between different populations of somatic cells in the fetal ovary, and therefore the genes and molecular events driving differentiation of ovarian cell types and their organization into functional ovaries remain unclear.

In this study, we combined protein-coding and non-coding RNA microarray data to identify novel genes that are expressed at higher levels in the ovary compared to testis during early mouse gonad development. Utilising quantitative real-time RT-PCR (qRT-PCR) and high resolution *in situ* hybridisation analyses, we validated 24 new genes expressed preferentially in the developing ovary. Hence, this study reveals a suite of novel markers potentially capable of distinguishing different somatic cell populations in the early mouse ovary and identifies the earliest described markers of XX germ cell differentiation.

## Results

### Identification of Novel Protein-coding Genes Expressed in a Sexually Dimorphic Manner

In order to identify novel genes that are expressed at higher levels in the ovary compared to testis during mouse gonad development, we interrogated published microarray data from whole gonads at 14.5 days *post coitum* (dpc) (NCBI GEO database accession GSE5334 and GSE4818 and GUDMAP database at http://www.gudmap.org) as well as microarray data of isolated supporting cells at 13.5 dpc ([25]; GEO: GSE4928) using B statistics (score >2, n = 8864). To minimize the rate of false positives, sexually dimorphic gene profiles detected in this microarray were compared to other published microarray data [26]. Candidate genes were further filtered for novel sexually dimorphic genes by removing any genes previously reported, including known ovary-specifically expressed markers such as *Xist* [27] and *Fst* [28] and testicular markers *Sox10* [29] and *Gdnf* [30]. This analysis resulted in the identification of 13 genes (**Table S1**)–*Lrrc34* (leucine rich repeat containing 34), *Smc1b* (structural maintenance of chromosomes 1B), *Lypd6* (LY6/PLAUR domain containing 6), *Egfl6* (EGF-like-domain, multiple 6), *4930422N03Rik*, *Dmrtc1c1* (DMRT-like family C1c1), *D6Mm5e* (DNA segment, Chr 6, Miriam Meisler 5, expressed), *Ccdc41* (coiled-coil domain containing 41), *2610019F03Rik*, *Spdya* (speedy homolog A), *Magi2* (membrane associated guanylate kinase, WW and PDZ domain containing 2), *Slirk1* (SLIT and NTRK-like family member 1) and *Fam196b* (family with sequence similarity 196, member B)–which were predicted to be expressed at higher levels in the embryonic ovary as compared to the testis and have not been previously investigated during mouse gonad development.

**Figure 1:**
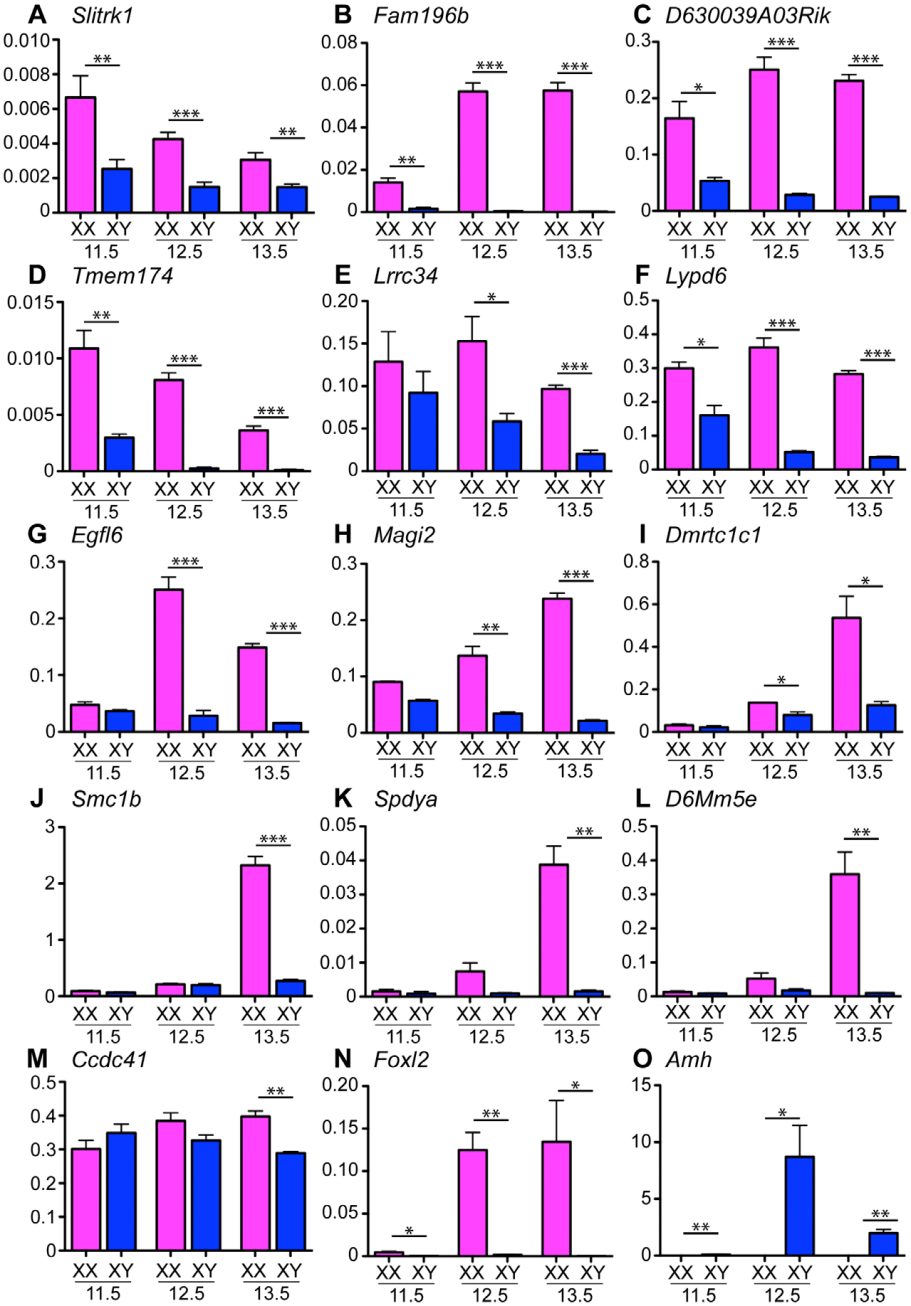
qRT-PCR validation of genes expressed in the developing ovary identified by microarray. qRT-PCR analysis of mRNA from isolated XX and XY gonads from 11.5, 12.5 and 13.5 dpc mouse embryos using gene-specific primers for *Slitrk1* (**A**), *Fam196b* (**B**), *D630039A03Rik* (**C**), *Tmem174* (**D**), *Lrrc34* (**E**), *Lypd6* (**F**), *Egfl6* (**G**), *Magi2* (**H**), *Dmrtc1c1* (**I**), *Smc1b* (**J**), *Spdya* (**K**), *D6Mm5e* (**L**), *Ccdc41* (**M**), *Foxl2* (**N**) and *Amh* (**O**) relative to *Sdha* (mean +SEM of at least three independent experiments; two-tailed, unpaired t-test; *p≤0.05, **p≤0.01, ***p≤0.001). Individual experiments were performed in triplicate on RNA obtained from pooled gonads from 3–4 littermates. All candidate genes were confirmed to be higher expressed in the ovary compared to testis at least at one of the developmental stages investigated. *Foxl2* served as control gene for ovary, *Amh* as control gene for testis samples.

To validate the dynamics and sex-specificity of expression of these genes, we performed quantitative real-time RT-PCR (qRT-PCR) of mRNA extracted from embryonic gonads from 11.5 to 13.5 dpc using the ovarian gene *Foxl2* (forkhead box L2) as an XX control ([31], Figure 1N) and the testicular gene *Amh* (encoding anti-Müllerian hormone) as an XY control ([32], Figure 1O). The qRT-PCR analysis revealed different temporal expression patterns of these genes that can be divided into 3 groups. Firstly, group A genes were expressed higher in the ovary as compared to the testis at all stages investigated. This class included *Slitrk1* (Figure 1A), *Fam196b* (Figure 1B), *D630039A03Rik* (Figure 1C), and *Tmem174* (Figure 1D). Secondly, group B genes displayed high expression at 11.5 dpc in XX and XY genital ridges but were subsequently down-regulated during testis differentiation with expression remaining at a high level in ovaries, similar to the expression profile described for *Wnt4* expression [33]. Two genes, *Lrrc34* (Figure 1E) and *Lypd6* (Figure 1F), showed this expression pattern. Group C genes were expressed at low levels in XX and XY genital ridges at 11.5 dpc before being up-regulated specifically in the developing ovaries; these included *Egfl6* (Figure 1G), and *Magi2* (Figure 1H). Some of the group C genes were characterized by no or very low expression in both sexes at 11.5 and 12.5 dpc, before being up-regulated in the ovary at 13.5 dpc such as *Dmrtc1c1* (Figure 1I), *Smc1b* (Figure 1J), *Spdya* (Figure 1K), and *D6Mm5e* (Figure 1L). The last gene, *Ccdc41* was expressed at similar levels at 11.5 and 12.5 dpc in gonads of both sexes, whereas at 13.5 dpc levels were significantly lower in the testis compared to ovary (Figure 1M).

### Identification of Novel Non-coding Genes Expressed in a Sexually Dimorphic Manner

**Figure 2:**
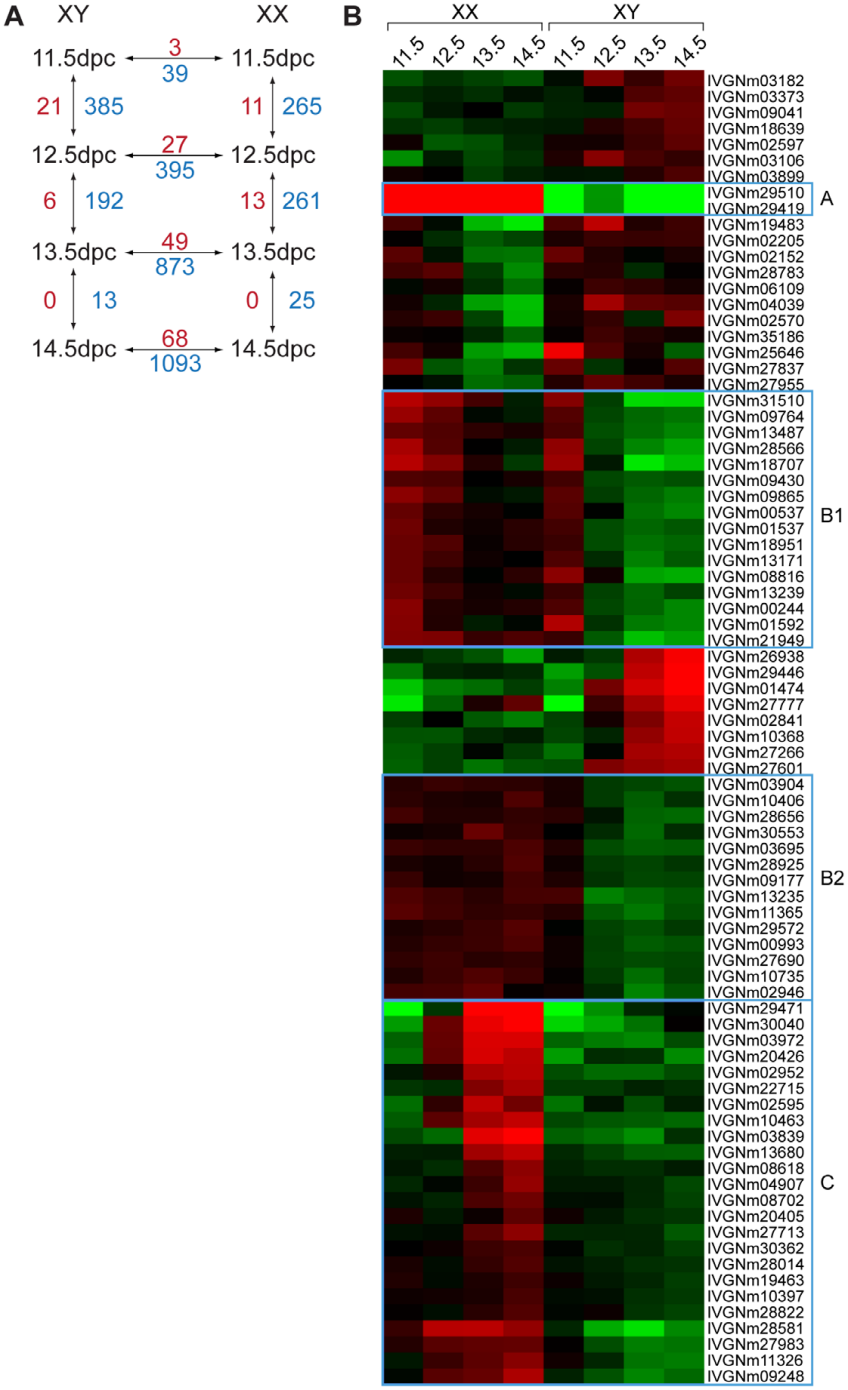
Sexually dimorphic expression of long ncRNAs during early mouse gonad development. (**A**) Microarray analysis identified a number of lncRNAs (red numbers) and mRNAs (blue numbers) to be differentially expressed during gonad development from 11.5 to 14.5 dpc. (**B**) Cluster analysis of microarray data identified four different classes (A, B1, B2 and C) of lncRNAs expressed specifically or preferentially in the developing ovary (annotations for lncRNA see Table S2).

To obtain a more complete set of genes expressed in the early ovary, we also employed non-coding RNA (ncRNA) microarrays for mouse embryonic gonads from 11.5 to 14.5 dpc. In addition to probes targeting ncRNAs, these microarrays also contained probes targeting 27,530 mRNAs. On these arrays, 7 of the 13 abovementioned protein-coding genes contained probes and confirmed the higher expression levels in the ovary compared to testis (**Figure S1**). Similar to protein-coding genes (**Table S2, second sheet**), many of the ncRNAs (**Table S2, first sheet**) displayed sexually dimorphic expression and/or differential gene expression at different stages during mouse gonad development (**Figure 2A**). Of the microarray probes targeting ncRNAs, a total of 82 showed sexually dimorphic expression between 11.5 and 14.5 dpc (**Figure 2B**). Of these, 56 had higher expression in the developing ovary and 26 showed higher levels in the developing testis. Similar to the mRNA candidates, cluster analysis showed that the “ovary-ncRNAs” (oncRNAs) were separated into different groups (A, B1, B2 and C, **Figure 2B**). Group A, consisting of only two probes, were highly specific to the ovary at all time points investigated. Both probes target *Xist*, a well-studied ncRNA involved in X chromosome inactivation [34]. Genes in group B showed expression during testis differentiation, with detectable levels at 11.5 dpc and down-regulation thereafter. This group was further subdivided into B1 and B2 based on the expression patterns during ovary development. Group B1 genes were expressed robustly in the ovary at 11.5 dpc and slowly down-regulated over the next three days of development, whereas group B2 genes maintained a moderate level of expression in the ovary throughout this period (**Figure 1B**). The last group, group C, contained genes whose expression was low in ovaries and testes at 11.5 dpc, with up-regulation in the developing ovaries thereafter (**Figure 1B**).

In this study, we focussed on the development of the early mouse ovary. Therefore, to prioritise oncRNA candidates for further characterization, we listed the transcripts exhibiting higher expression in ovaries compared to testes from 11.5 to 13.5 dpc (Table S3). The two top probesets (*oncRNA1* and *2*), which displayed high expression in the developing ovary at all three stages, target *Xist* and were therefore excluded from in depth analysis. For further validation using qRT-PCR of embryonic gonad mRNA from 11.5 to 13.5 dpc, we chose the top 11 candidates (Table S3, marked in red) and gave them code numbers to avoid confusion due to the high similarity and complexity of the “AK” numbers: *AK019493* (designated in this study as *oncRNA3*), *AK036014* (*oncRNA4*), *AK046039* (*oncRNA5*), *AK015184* (*oncRNA6*), *AK017289* (*oncRNA10*), *AK156088* (*oncRNA11*), *AK034059* (*oncRNA12*), *AK015136* (*oncRNA13*), *AK021294* (*oncRNA15*), *AK014986* (*oncRNA16*), and *AK015693* (*oncRNA17*). We were not able to design qRT-PCR primers to detect *AK044909* (*oncRNA14*) unequivocally, because this transcript completely overlaps with a predicted protein-coding gene, *ENSMUST00000070150*.

**Figure 3:**
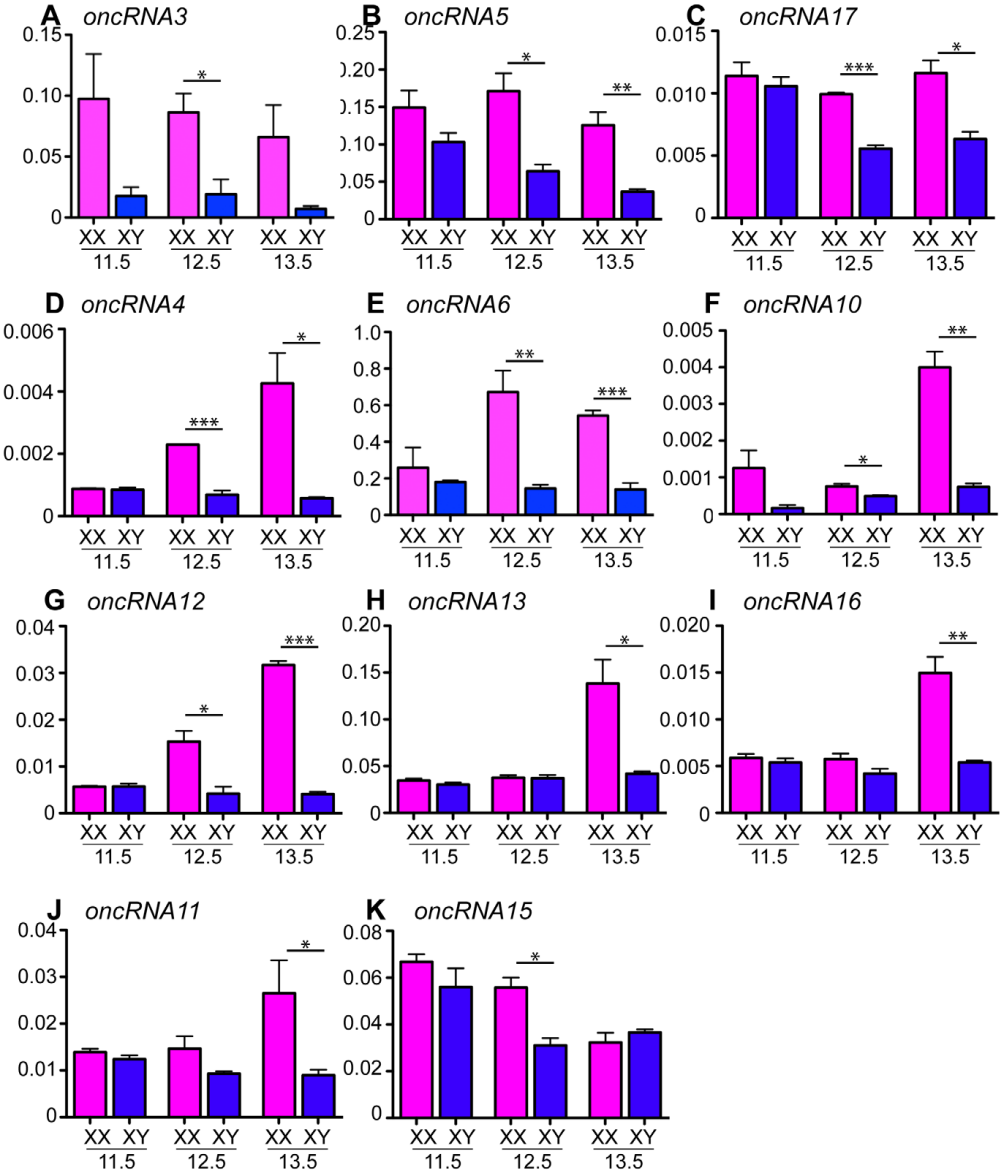
qRT-PCR validation of ncRNAs preferentially expressed in the developing ovary identified by microarray. qRT-PCR analysis of isolated XX and XY gonads from 11.5, 12.5 and 13.5 dpc mouse embryos using gene-specific primers for *oncRNA3* (**A**), *oncRNA5* (**B**), *oncRNA17* (**C**), *oncRNA4* (**D**), *oncRNA6* (**E**), *oncRNA10* (**F**), *oncRNA12* (**G**), *oncRNA13* (**H**), *oncRNA16* (**I**), *oncRNA11* (**J**), and *oncRNA15* (**K**) relative to *Sdha* (mean +SEM of at least three independent experiments; two-tailed, unpaired t-test; *p≤0.05, **p≤0.01, ***p≤0.001). Individual experiments were performed in triplicate on RNA obtained from pooled gonads from 3–4 littermates. All candidate genes were confirmed to be ovary-enriched in their expression.

As expected from the cluster analysis, the temporal expression of these transcripts fell into the three groups with *oncRNA3* being the only ncRNA in group A (Figure 3A). Two ncRNAs, *oncRNA5* and *oncRNA17*, belonged to group B (Figure 3B and C) and the majority, including *oncRNA4*, *oncRNA6*, *oncRNA10*, *oncRNA12*, *oncRNA13*, *oncRNA16* and *oncRNA11* to group C (Figure 3D, E, F, G, H, I, J). *oncRNA15* displayed an unusual expression pattern with high expression in testis and ovary at 11.5 dpc, followed by a reduction in expression levels at 12.5 dpc in testes but not ovaries, and a subsequent reduction in ovaries at 13.5 dpc. This profile resulted in a significantly higher expression in the ovary compared to testis transiently at 12.5 dpc (Figure 3K).

### Germ Cell-specific Gene Expression Pattern

**Figure 4:**
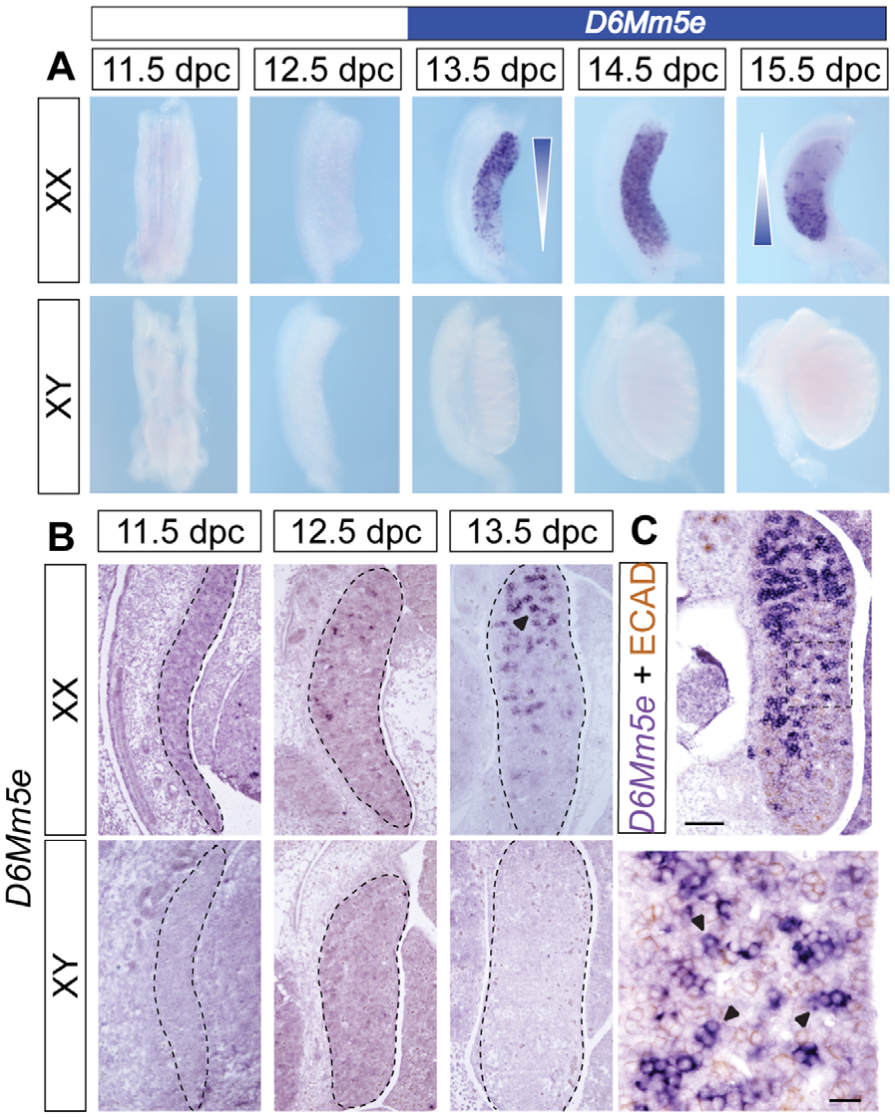
Temporal and spatial expression analysis of *D6Mm5e*. Whole mount ISH of mouse embryonic XX and XY gonads from 11.5 to 15.5 dpc (**A**) and ISH with sagittal section of mouse embryonic XX and XY gonads from 11.5 to 13.5 dpc, (**B**), showing wave-like upregulation of *D6Mm5e* reminiscent of PGC (arrowhead) entry into meiosis. Scale bar, 100 µm. (**C**) *D6Mm5e* section ISH hybridization (purple staining) followed by IHC for the germ cell marker E-cadherin (ECAD, brown staining) of 13.5 dpc mouse ovaries confirmed *D6Mm5e* expression in XX PGCs (arrowheads). Scale bars, 100 µm (low magnification, top panel) and 20 µm (high magnification, bottom panel).

Having verified the sexually dimorphic expression of these 23 genes by qRT-PCR, we next performed whole-mount and section ISH on a subset in order to identify the spatial and cellular expression patterns of these genes. At the early stages of ovary development, primordial germ cells (PGCs) are distinguishable by their round morphology, whereas it is not possible to distinguish between different somatic cell types. Genes expressed predominantly by PGCs are visualised as a punctate staining pattern in whole-mount ISH and as large, ring-shaped staining domains by section ISH. To further confirm cell type specific expression, we interrogated two different, previously published microarray data [35], [36]. Firstly, a study using *W^v^/W^v^* mutant mouse gonads that lack germ cells ([36]; **Figure S2**) and secondly, transcriptional profiles of sorted gonadal cells ([35]; Table S1). In addition, we performed section ISH on 13.5 dpc XX wild-type embryos and *W^e^/W^e^* embryos that lack germ cells ([37]; **Figure S3**). Five genes - *D6Mm5e*, *Dmrtc1c1*, *Spdya*, *Lrrc34* and *oncRNA6* - were suggested to be expressed in PGCs by the microarray as well as ISH analysis (**Figure 4** **,** **5** **,** **6** and **Figure S2, S3, S4**) in the developing gonads from 11.5 to at least 13.5 dpc.

**Figure 5:**
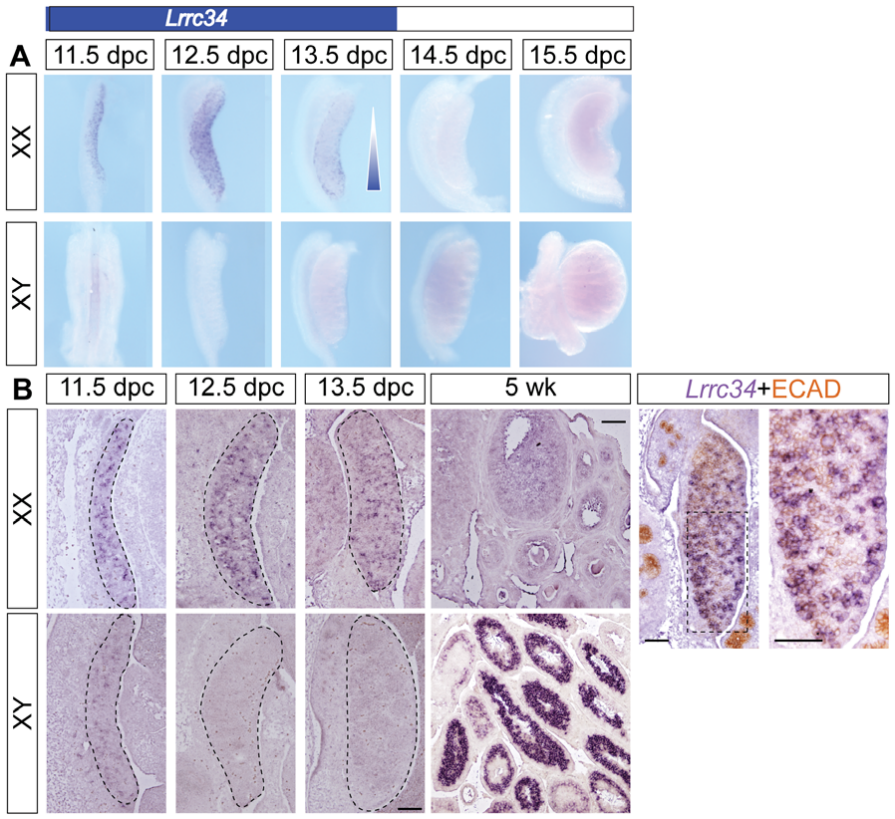
*Lrrc34* expression during gonad development. Whole-mount ISH (**A**) of XX and XY mouse embryonic gonads from 11.5 to 15.5 dpc and ISH (**B**) with sagittal section of XX and XY mouse embryos from 11.5 to 13.5 dpc, 5 weeks (wk) postnatal mouse ovaries and testes as well as section ISH (purple staining) followed by IHC (brown staining) for the germ cell marker E-cadherin of 13.5 dpc ovaries (**B,** last panel) demonstrated that *Lrrc34* is XX germ cell-specifically expressed before 13.5 dpc. *Lrrc34* expression is down-regulated in an anterior-to-posterior wave from 13.5 dpc onwards (**A**, **B**). Scale bars, 100 µm.

**Figure 6:**
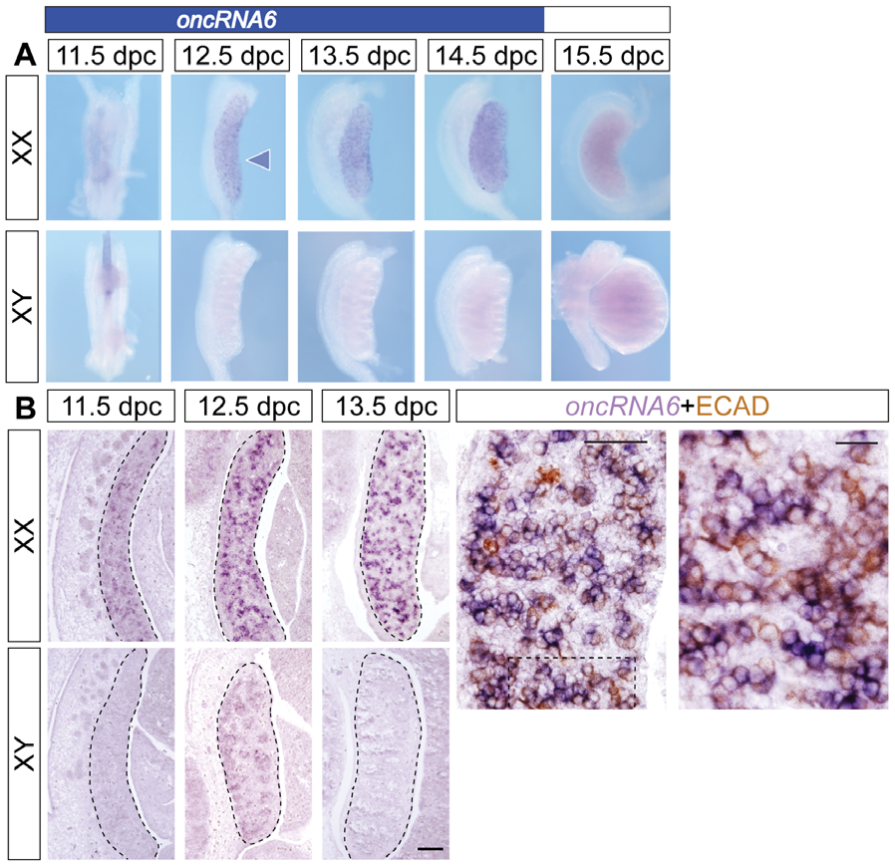
oncRNA6 expression during gonad development. Whole-mount ISH (**A**) of XX and XY mouse embryonic gonads from 11.5 to 15.5 dpc and ISH with sagittal section (**B**) of XX and XY mouse embryos from 11.5 to 13.5 dpc, as well as section ISH (purple staining) followed by IHC (brown staining) for the germ cell marker E-cadherin of 13.5 dpc ovaries (**B,** last panel) demonstrated that *oncRNA6* is XX germ cell-specifically expressed before 13.5 dpc. Scale bars, 100 µm (**B**, first three panels), 50 µm (**B**, ISH/IHC low magnification), 20 mm (**D**, ISH/IHC high magnification).

Three of the five genes, *D6Mm5e*, *Dmrtc1c1*, *Spdya,* belonged to the last class of temporal expression pattern characterized by up-regulation in the developing ovary at 13.5 dpc. ISH showed that these genes were expressed in a gradient with high expression at the anterior and low or undetectable level at the posterior pole of the developing ovary at 13.5 dpc, with expression extending through the whole ovary by 14.5 dpc (Figure 4A, B for *D6Mm5e*, Figure S4A, B for *Dmrtc1c1*, Figure S4B, C for *Spdya*), reminiscent of the wave of PGC entry into meiosis in the ovary [15], [16]. PGC-specific expression was further corroborated by microarray data (Figure S2; [36]) as well as section ISH (Figure 4C, S4C, purple staining) combined with immunohistochemistry (IHC) for E-cadherin (Figure 4C, brown staining), a protein known to be expressed by germ cells [38] or immunohistochemistry for the somatic cell marker FOXL2 (Figure S4C, brown staining). Based on temporospatial criteria, these genes appear to mark early meiotic germ cells.

Interestingly, two of the genes that were primarily expressed by XX germ cells, *Lrrc34* (Figure 5A, B) and the long ncRNA *oncRNA6* (Figure 6A, B), were detected in a sexually dimorphic pattern in PGCs some two days before apparent germ cell differentiation at 13.5 dpc. Whole-mount (Figure 5A) and section ISH (Figure 5B) showed that *Lrrc34* was expressed in XX PGCs from 11.5 dpc with expression starting to decrease at 13.5 dpc in an anterior-to-posterior wave. At 5 weeks postnatally, no *Lrrc34* expression was detected in the ovary. In contrast, *Lrrc34* was detected in the testis in round spermatids and, to a lesser extent, in late spermatocytes. No expression was detectable in spermatogonia, early prophase spermatocytes, elongating spermatids, Sertoli cells or interstitial cells (Figure 5B). The long ncRNA *AK015184* (*oncRNA6*) was detected in XX but not XY PGCs from 11.5 dpc until at least 14.5 dpc (Figure 6A, B). Section ISH followed by IHC for E-Cadherin suggested that *oncRNA6* was expressed by most, but possibly not all XX PGCs (Figure 6B, last panel). To our knowledge, *Lrrc34* and *oncRNA6* therefore represent the first genes to be identified as sexually dimorphic in PGCs at 11.5 and 12.5 dpc, before XX germ cells enter meiosis.

### Novel Ovarian Somatic Cells Specifically Expressed Genes

**Figure 7:**
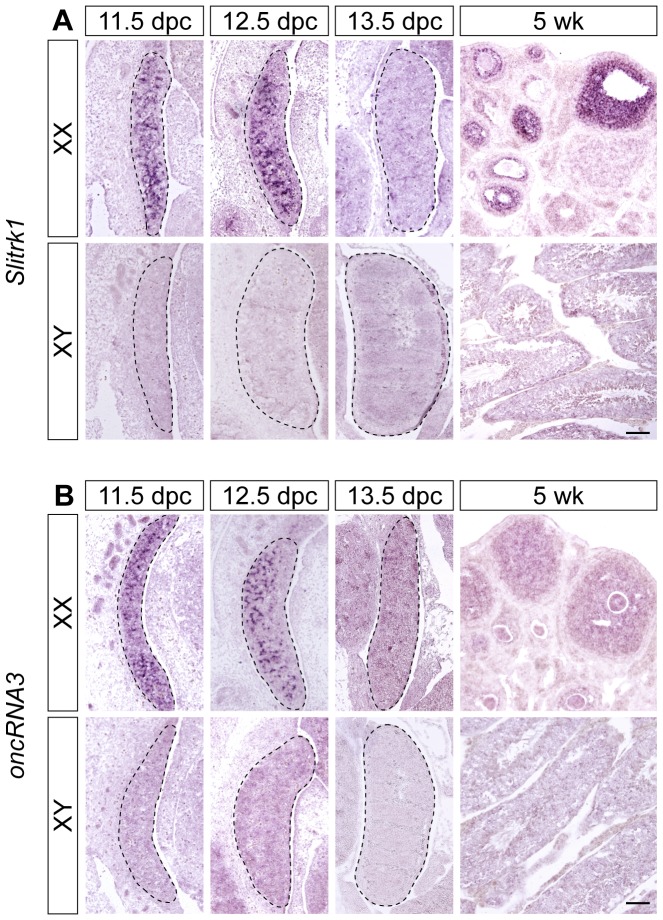
Expression analysis of somatic cell genes. ISH with sagittal section of XX and XY mouse embryos from 11.5 to 13.5 dpc, 5 weeks (wk) postnatal mouse ovaries and testes showed that *Slitrk1* (**A**) and *oncRNA3* (**B**) are expressed in ovarian somatic cells at 11.5 and 12.5 dpc. *Slitrk1* is also expressed in the granulosa cells of the mature ovary. Scale bars, 100 µm.

We next verified the sexually dimorphic candidates predominantly expressed by somatic cells using section ISH of mouse embryos from 11.5 to 13.5 dpc. Two of these genes, *Slitrk1* (**Figure 7A**) and *oncRNA3* **(** **Figure 7B**), were only detectable in the ovary at 11.5 and 12.5 dpc before decreasing to undetectable levels by 13.5 dpc. *Slitrk1* was detected at 5 weeks postnatally, with the main expression restricted to granulosa cells in the ovary (**Figure 7A**). Neither of these genes were expressed at detectable levels in the testis at any of the stages analysed (**Figure 7A**). In addition, we detected expression in ovarian somatic cells for *Lypd6*, *Magi2* and *Egfl6* (**Figure S5**).

### Genes Predominantly Expressed in Ovarian Somatic Cells Occupy Different Domains

**Figure 8:**
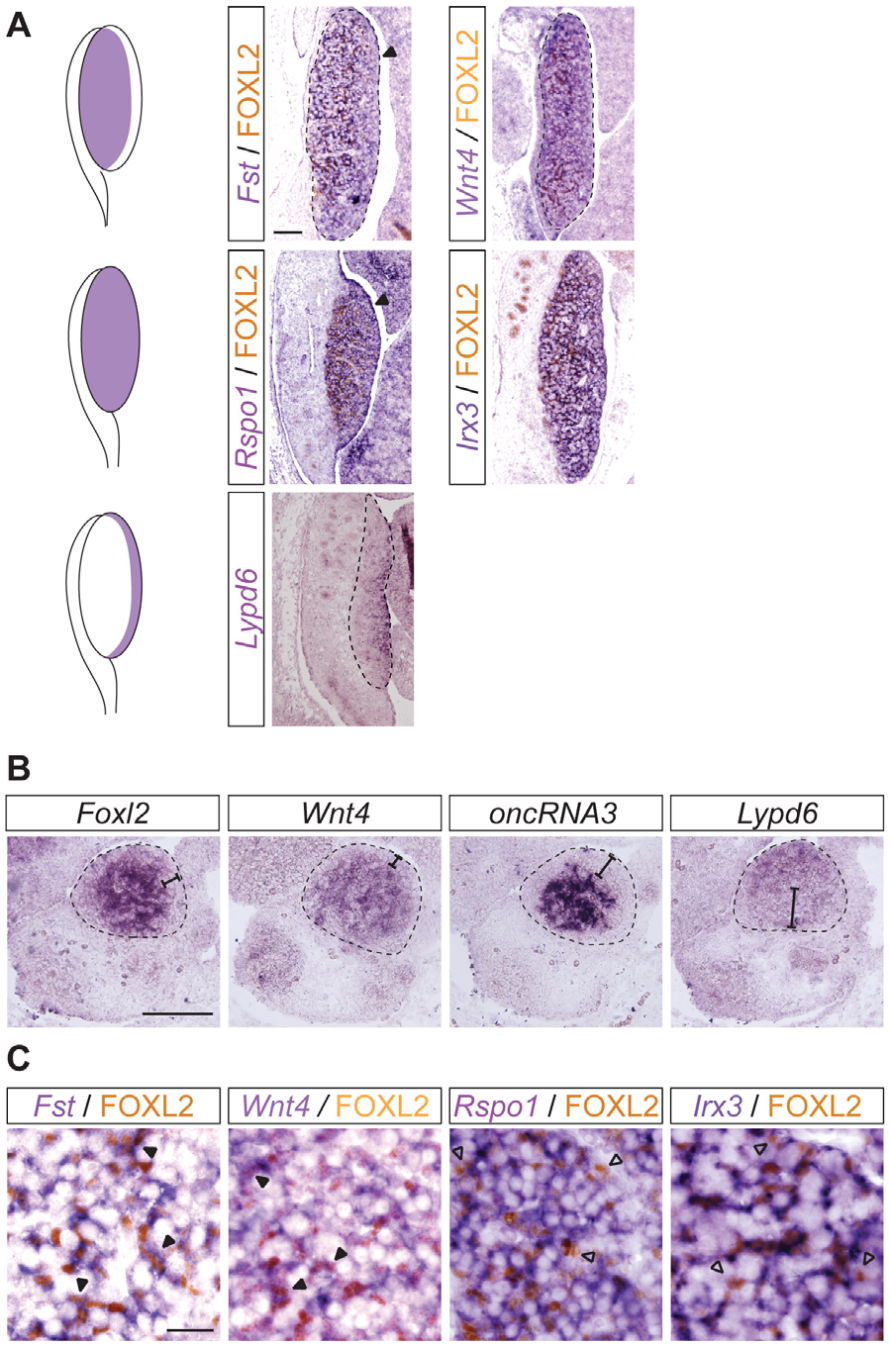
Molecular compartmentalization of the early embryonic ovary. (**A**) ISH with sagittal section of 13.5 dpc XX embryos for *Fst*, *Wnt4*, *Rspo1* and *Irx3* followed by IHC for FOXL2 (first and second panel) and section ISH for *Lypd6* (third panel) identified three different expression domains of somatic cell genes as represented in the schematic on the left of each panel. Scale bar, 100 µm. (**B**) ISH with transverse sections of 12.5 dpc XX embryos for *Foxl2*, *Wnt4*, *oncRNA3* and *Lypd6* showed the extent to which these genes were expressed in the mesonephric and coelomic domains respectively. (**C**) High magnification of ISH with sagittal sections of 13.5 dpc XX embryos for *Fst*, *Wnt4*, *Rspo1* and *Irx3* followed by IHC for FOXL2 suggested that *Fst* and *Wnt4*, but not *Rspo1* and *Irx3*, are co-expressed to a large extent with FOXL2. Scale bar, 100 µm (**A** and **B**), 20 µm (**C**).

Having identified new genes with expression detected in ovarian but not testicular somatic cells we next analysed the spatial expression patterns of novel (**Figure 7** **,** **8A and S** **5**) and known (**Figure 8A**) genes with high expression in the ovary. Section ISH for *Foxl2*, *Fst*, *Wnt4*, *Rspo1*, *Irx3* (Iroquois related homeobox 3), *Slitrk1*, *oncRNA3*, *Lypd6*, *Magi2* and *Egfl6* on sagittal sections of 13.5 dpc mouse embryos revealed that the expression of these genes can be categorized into three different spatial expression patterns. The majority of genes including *Fst* and *Wnt4* (**Figure 8A**), *Slitrk1* and *oncRNA3* **(** **Figure 7**), *Egfl6* (**Figure S5**) and *Foxl2* (data not shown) displayed high expression levels from the mesonephros (future ovarian medulla) up to a few cell layers from the coelomic epithelium (future ovarian cortex; **Figure 8A**, upper panel). The second group of genes encompassing two of the genes investigated, *Rspo1* and *Irx3*, were expressed throughout the developing ovary including the coelomic epithelium (**Figure 8A**, middle panel). In contrast, genes of the third category including *Lypd6* (**Figure 8A**) and *Magi2* (**Figure S5**) as well as *Bmp2* [28], displayed higher expression at the side of the coelomic epithelium and no or only weak expression close to the mesonephros. We have previously reported similar spatial gradients of gene expression in the early developing testis [39].

To further characterize these expression domains, we performed two types of experiments. Firstly, ISH on transverse section of 12.5 dpc XX embryos revealed the extent to which these genes were expressed towards the coelomic epithelium and the mesonephros respectively (**Figure 8B**). Interestingly, *Foxl2* and *Wnt4* were not detectable in the coelomic epithelium and a maximum of one to two cell layers below the epithelium (**Figure 8B**, first and second panel), whereas *oncRNA3* was not detectable within several cell layers from the coelomic epithelium (**Figure 8B**, third panel), and *Egfl6* was restricted to just a few cell layers adjacent to the mesonephros (**Figure S5**). In comparison, *Lypd6* was expressed in the coelomic epithelium and several adjacent cell layers, but not in cells bordering the mesonephros (**Figure 8B**, fourth panel).

Lastly, we performed co-expression analysis by combining ISH with IHC for FOXL2, a known ovary-determining gene [7]–[9], and an example of the first category (not expressed in the coelomic epithelium plus one or two subjacent cell layers; **Figure 8A, C**). Other genes of the first category, such as *Fst* and *Wnt4*, displayed extensive overlap with FOXL2 (**Figure 8C**, first and second panel), whereas there appeared to be less co-expression with genes of the second and third category (expressed throughout the developing ovary including the coelomic epithelium and expressed in the coelomic epithelium and cell layers below, respectively), for example *Rspo1* and *Irx3* (**Figure 8C**, third and fourth panel).

## Discussion

Our knowledge about the molecular and cellular basis of early mouse ovarian development is limited. A number of studies have identified genes that are expressed at higher levels in the developing ovary compared to the testis [5], [26], [35], [40], [41], however only few genes have been shown to play a role in ovary differentiation (for review see: [18]). These genes can be broadly categorized into somatic- and germ cell-specifically expressed genes. In this study we have identified additional protein- and non-coding genes that are expressed at higher levels in the ovary compared to testis during gonad differentiation. We describe distinct categories for these genes based on their temporospatial expression domains. Specifically, they have been classified into i) genes detectable in XX, but not XY, germ cells before entry into meiosis; ii) meiosis-associated XX germ cell genes; iii) genes detectable in somatic cells in a gradient with higher expression at the mesonephric side; iv) somatic cell genes detectable evenly throughout the ovary; and v) somatic cell genes that are detectable in and near the coelomic epithelium (observed expression pattern summarized in **Figure S6**).

### Long ncRNAs are Expressed Sexually Dimorphic

Non-coding RNAs can be loosely grouped into two classes based on transcript length, namely small and long ncRNAs. Recent research has focused mainly on small RNAs, with microRNAs (miRNAs) being the best known of these. Accordingly, miRNA expression and function has been studied in the postnatal ovary [42]–[44]. In contrast, less is known about long ncRNAs (lncRNAs). Generally, lncRNAs appear to be expressed at lower levels than protein-coding genes [45]–[47], and some are tissue-specifically expressed [48]. Here, we analysed for the first time the expression of lncRNAs during early ovarian differentiation using microarrays. In addition to the well-characterized lncRNA *Xist* [34], we identified many lncRNAs displaying sexually dimorphic expression during mouse gonad development and verified the expression of some of candidates that had higher expression levels in the ovary compared to testis by qRT-PCR and ISH.

The challenge now is to elucidate the role of these ncRNAs during ovarian development. The small number of lncRNAs that have been functionally characterized to date often regulate the expression of nearby genes through a variety of different biological processes such as epigenetics, alternative splicing, nuclear import, structural integrity, modulators of RNA degradation and precursors for small RNAs [49]–[54]. We have identified *oncRNA3* to be expressed predominantly by ovarian somatic cells. *oncRNA3* is antisense to the gene encoding pantothenate kinase 1 (*Pank1*), suggesting that *Pank1* is regulated by this lncRNA. However, qRT-PCR analysis showed that *Pank1* expression is not sexually dimorphic (data not shown) and appears to have no function during gonad development [55]. Hence, *oncRNA3* must play a different role during ovarian development.

One of the major signalling pathways driving ovarian differentiation is WNT4/RSPO1 signalling [6], [10], [11], [56]. The loss of this signalling pathway in XX gonads results in partial ovary-to-testis sex reversal [6], [10], [11] and the constitutive activation in XY gonads to complete testis-to-ovary sex reversal [56], demonstrating the importance of this signalling pathway. It would be interesting to test if the expression of any of the ncRNAs identified here are affected by WNT signalling. The expression profiles of protein-coding genes in wild-type vs. *Wnt4*-knockout gonads has been analysed previously [57]. The only gene from our dataset whose expression was found in that study to be altered in *Wnt4*-null gonads is *Smc1b* (former *Smc1/2*). However, the method used (Operon Mouse Genome oligo set) and time points investigated (12.0 dpc) were limited, leaving the possibility open that the expression of other, here identified, genes is regulated by WNT signalling.

### XX and XY Germ Cells are Sexually Dimorphic Before Entry into Meiosis

One of the first morphological events during ovary development is the entry into meiosis of PGCs at 13.5 dpc in an anterior-to-posterior wave [15], [16], which is followed by the onset of expression of meiosis-related genes such as *Stra8* and *Scp3*, as well as the down-regulation of pluripotency markers, e.g. *Oct4* [15], [58]. We describe here the expression of four other genes (*D6Mm5e*, *Spdya*, *Smc1b* and *Dmrtc1c1)* with expression patterns reminiscent of the wave of XX germ cell entry into meiosis. A role in meiosis has been implicated previously for the first three genes, *D6Mm5e*, *Spdya*, and *Smc1b* [59]–[61]. In contrast, little information exists for the X-chromosomal gene *Dmrtc1c1* (also known as *Dmrt8.3*). This gene belongs to the DM gene family encoding transcription factors that contain a DM domain. *Dmrtc1c1* was first described in humans as *DMRT8*, however it contains a stop codon 3′ of the DM domain, suggesting it might not encode an active protein in human [62], [63]. In mouse, rat and rabbit three *Dmrt8* genes (*Dmrt8.1*, *Dmrt8.2* and *Dmrt8.3*) exist in a cluster on the X chromosome [64]. In mouse, *Dmrt8.1* and *Dmrt8.2* have been shown to be testis-specifically expressed, whereas no expression was detected for *Dmrt8.3* (*Dmrtc1c1*; [64]). We found that *Dmrt8.3* (*Dmrtc1c1*) expression is ovary-specific, with the main expression in XX germ cells from 13.5 dpc onwards, suggesting a role in meiosis. However, it still needs to be determined whether this member of the DM gene family is translated or functions as a non-coding RNA.

It is believed that entry into meiosis in mammals is a non-cell autonomous process, i.e. the sex chromosome composition of PGCs is irrelevant for this differentiation process, which is induced by factors from the environment [65]. PGCs in an ovary will enter meiosis at 13.5 dpc [15], [16], whereas PGCs in a testis arrest in mitosis [17]. To date, only one study has reported sexually dimorphic gene expression in PGCs before their differentiation; the microRNA *miR-302* is XY-specifically expressed at 8.5 and 9.5 dpc [66]. Interestingly, we found that two other genes, the X-chromosomal lncRNA *oncRNA6* and the autosomal gene *Lrrc34*, were detected in XX, but not XY, germ cells before they enter meiosis. Neither gene has been characterized to date, and their functions are unknown. However, the expression of *Lrrc34*, encoding a protein predicted to be involved in protein-protein interaction, is down-regulated as XX germ cells enter meiosis, potentially implicating this gene in preparing PGCs for this differentiation process. In the testis, *Lrrc34* is expressed postnatally in late spermatocytes and round spermatids, i.e. during and after meiosis, suggesting a different role of *Lrrc34* during oogenesis and spermatogenesis.

### Molecular Regionalization of the Embryonic Mouse Ovary

The ovary in many species, including most vertebrates, is regionalized into a highly vascularised medulla and a cortical region in which the germ cells reside [67]. In mouse, this regionalization is not obvious at a morphological level until shortly before birth. At 16.5 dpc, germ cells that reside in the medullary region of the mouse ovary go into apoptosis followed by the deposition of a laminin-rich boundary that divides the ovary into a defined germ cell-containing cortex and germ cell-free medulla [28]. In contrast, a molecular regionalization based on gene expression in somatic cells is established as early as 12.5 dpc, with *Bmp2* expression in the presumptive cortical region and *Wnt4* and *Fst* in the medulla [28]. We identified two other genes, *Lypd6* and *Magi2*, that are expressed in the coelomic domain together with *Bmp2*. LYPD6 is a cytoplasmic protein that belongs to the Ly-6 superfamily. The only function identified to date is its ability to inhibit AP1-induced gene expression [68]. In contrast, MAGI2, also known as S-SCAM (synaptic scaffolding molecule) belongs to the membrane-associated guanylate kinase superfamily [69]. It is believed that its main function is as a scaffold protein to assemble and anchor signalling proteins including ß-catenin [70]. ß-catenin is a key pro-ovarian and anti-testis signalling molecule [56], suggesting that *Magi2* has an important role during ovarian differentiation.

By far the majority of the genes we investigated, including *Slitrk1*, *Egfl6* and the lncRNA *oncRNA3*, were detectable in a similar pattern to *Wnt4* and *Fst*, with the highest expression along the mesonephros and low or no expression within the coelomic domain. These genes appeared, in most cells, co-expressed with FOXL2, which might not be surprising since *Foxl2* expression has been shown to mark the precursors not only of granulosa but also of theca cells [71]. Further experiments are required to distinguish between the different somatic cell lineages in early ovary differentiation.

SLITRK1 is a transmembrane protein that controls neurite outgrowth [72]. Its expression has been documented in detail only during brain development, and mutations in humans have been associated with the developmental neuropsychiatric disorder Tourette syndrome [73], [74]. The present study is the first to describe *Slitrk1* expression during gonad development. Accordingly, *Slitrk1*-null mice have been characterized regarding their behavioural abnormalities but not with respect to fertility or reproduction [75]. It would be interesting to determine whether loss of *Slitrk1* results in an ovarian phenotype. Similarly, the role of EGFL6, a member of the epidermal growth factor superfamily, during ovarian development is unclear. However, its expression is upregulated in ovarian cancer [76], marking *Egfl6* as an interesting candidate for ovarian differentiation.

### Conclusions

In conclusion, this study identified several genes that were preferentially expressed in ovary relative to testis, and may therefore play a role in ovarian differentiation. The observation of different classes of genes based on their temporospatial pattern of expression is an important step towards characterization of the various somatic cell precursors in the fetal ovary. Further co-localization studies are likely to reveal groups of genes that are consistently co-expressed in specific subsets of ovarian cells, which can be used as markers to track the origins, movements, interactions and fates of these different precursor cells. This knowledge in turn will play an important role in efforts to understand the etiology of female infertility, ovarian failure and ovarian cancer, and potentially also suggest novel ways to manage these disorders.

## Methods

### Mouse Strains

Embryos were collected from timed matings of outbred CD1 strain mice and the *c-Kit^We/+^* inbred strain [37], with noon of the day on which the mating plug was observed designated 0.5 days *post coitum* (dpc). For more accurate staging of embryos up to 12.5 dpc, the tail somite (ts) stage was determined by counting the number of somites posterior to the hind limb [77]. Using this method, 10.5 dpc corresponds to approximately 8 ts, 11.5 dpc to 18 ts, and 12.5 dpc to 30 ts. The presence of the Y chromosome was determined by PCR using *Zfy* primers. Protocols and use of animals were approved by the Animal Welfare Unit of the University of Queensland (approval # IMB/131/09/ARC), which is registered as an institution that uses animals for scientific purposes under the Queensland Animal Care and Protection Act (2001).

### Antibodies

Primary antibodies and dilutions used were: rabbit anti-SRY [78], [79] and rabbit anti-SOX9 [29], [79] at 1∶100; and rabbit anti-FOXL2 at 1∶500. Secondary antibodies used were anti-rabbit IgG (Invitrogen) and anti-chicken IgG (Sapphire Bioscience) conjugated to biotin.

### Data Analysis

Microarray developmental time course of mouse XY and XX whole gonads from GUDMAP (GEO: GSE5334, GSE4818; www.gudmap.org) was used to identify sexually dimorphic genes/probesets at 14.5 dpc using B-statistics analysis (*Limma* package) in the R statistical program [80]. Probesets with B-score >2 were selected, which is associated with a 90% probability of being truly expressed. Genelists were further analysed using Genespring GX 7. Candidates expressed with a ≥2-fold difference in XX vs. XY at 14.5 dpc were selected (Table S1). To ensure specificity, gene profiles were compared to a dataset from a previously published time course [26] (GEO: GSE6916). Genes that were previously identified as sexually dimorphic genes were excluded by using literature curation databases such as the Ingenuity Pathway Analysis (Ingenuity) program and annotations from the Mouse Genome Consortium (MGI). Only novel ovary-enriched genes were selected for validation.

### Non-coding RNA Microarray

Differential expression of long non-coding and messenger RNAs was performed using the NCode Mouse array (Life Technologies). The microarrays contained probes to target 8,061 long ncRNAs and 27,530 mRNAs. Gonad samples were collected at the specified stages and snap-frozen. Total RNA from all samples was prepared in parallel using RNeasy Micro Kit (Qiagen) from pooled testes and ovaries from 11.5 dpc to 14.5 dpc with 15 genital ridges per pool at 11.5 dpc and 12 to 14 gonad pairs per pool at 12.5 to 14.5 dpc. The quality of the RNA was analysed using a BioAnalyzer and was of high quality (RIN score >7). Two biological replicates for each stage of testis and ovary formation (12 samples) containing 5 µg labelled RNA were hybridised to individual dual colour NCode microarrays. Blocking, hybridization and washing were performed according to the manufacturer’s instructions (Agilent Technologies). The testis and ovary samples were hybridised independently using a “box” experimental design with dye swaps, where each time point was hybridised together with every other time point. Slides were scanned at 5 µm resolution using a DNA microarray scanner (Agilent Technologies).

Data was extracted using Agilent Feature Extraction software and analysed using the Linear Models for Microarray Data (LIMMA) software package via the R Project for Statistical Computing (www.r-project.org). Data was background-corrected, normalised both within and between arrays [81], and differential expression analysis was performed by fitting a linear model of the data to the experimental design matrix and then calculating Bayesian statistics (B statistics; posterior log odds) adjusted for multiple testing using Benjamini-Hochberg analysis [80]. This approach considers all the microarray data as an interrelated set and in doing so removes any individual outliers that may arise due to poor spot quality on an individual array. The microarray data conforms with MIAME guidelines and raw and normalized microarray data has been submitted to ArrayExpress Data Warehouse (EMBL-EBI; Accession ID: pending). Criteria used for selection were a B-statistic (log odds score) of three or greater, a fold-change of two or greater and a log2 mean expression level of six or greater.

Clustering was performed with Gene Cluster 3.0. Data was adjusted by log transformation and gene expression values were centred around the mean. Genes were organized by k-Means clustering (k = 10) using the euclidean distance similarity metric. The clustered data was visualised using Java Treeview.

### Real Time RT-PCR (qRT-PCR)

qRT-PCR was performed as described previously [82]. Primer sequences are listed in **Table S4**. For each gene, data sets were analysed for statistically significant differences between XX and XY expression levels using a two-tailed, unpaired t-test with confidence intervals set at 95%.

### In Situ Hybridisation (ISH)

Primer design and riboprobe generation has been described in detail previously [83] and is available on the GUDMAP website (http://www.gudmap.org/Research/Protocols/Little.html). In brief, microarray probe sets of the Affymetrix 430.2 were mapped to FANTOM3 cDNA clones set and mapped to the mouse genome (mm9) to ensure specificity of primers. PCR primers were designed to amplify a 500–800 bp region of each gene using *Primer3*. Candidate primers were checked for specificity of amplification of PCR product using the UCSC Genome Browser “*in-silico* PCR” prediction tool.

Whole mount ISH was performed as described on the GUDMAP gene expression database (http://www.gudmap.org/Research/Protocols/Little.html). The annotated expression patterns and WISH images for the following genes are available via the GUDMAP website (www.gudmap.org); *2610019F03Rik*, *4930422N03Rik*, *Ccdc41*, *D6Mm5e*, *Dmrtc1c1*, *Egfl6*, *Lrrc34*, and *Spdya*. All whole-mount tissues were photographed manually on a 1.5% agarose gel dish, using Nikon SMZ1500 stereomicroscope system with a Nikon DXM1200f, 12-megapixel digital camera, and ACT-2U Image Application Software. Section ISH was performed as described previously [84] and imaged with an Olympus BX-51 microscope.

### Double Staining

Simultaneous detection of RNA and protein in tissue sections was carried out by section *in situ* hybridization as described above followed by immunohistochemistry (IHC). Following the ISH colour reaction, sections were re-fixed with 4% PFA/PBS for 10 min at room temperature, and treated for antigen retrieval by boiling the samples for 8 min in antigen unmasking solution (Vector) and allowing them to cool down to room temperature for 1 h. To block endogenous peroxidase, slides were incubated in 3% H_2_O_2_ for 20 min. After washing in PBS, sections were blocked in 10% heat-inactivated horse serum (Gibco) for 1 h at room temperature, followed by incubation with primary antibody diluted in blocking solution overnight at 4°C. The slides were then washed three times with PBS and incubated with secondary antibody at a dilution of 1∶1000 for 1 h at room temperature. After 3 washes in PBS, colour reaction was performed using the Vectastain DAB kit following the manufacturer’s instructions. The slides were mounted in aqueous mounting medium (Vector laboratories) and imaged with an Olympus BX-51 microscope.

## Supporting Information

Figure S1 **Expression profiles of differentially expressed genes.** Normalized microarray expression data (mean ± standard deviation of four independent pools of isolated gonads) of differentially expressed mRNAs, *Lrrc34* (**A**), *D6Mm5e* (**B**), *Spdya* (**C**), *Smc1b* (**D**), *Egfl6* (**E**), *Magi2* (**F**), and *Lypd6* (**G**).
(TIF)

Figure S2 **Expression analysis in** ***W^v^/W^v^*** **mutant mouse ovaries.** Microarray analysis of candidate genes comparing expression levels in wild type (solid pink bars) and mutant (cross-hatched pink bars) at 12.0, 14.0 and 16.0 dpc suggested that the expression of *oncRNA6, D6Mm5e, Spdya, Dmrtc1c1, Lrrc34, Smc1b* and *Ccdc41* is dependent on the presence of germ cells. y-axis, relative expression levels on a Log2 scale [36].
(TIF)

Figure S3 **Expression analysis in** ***W^e^/W^e^*** **mutant mouse ovaries.** ISH with sagittal section of 13.5 dpc XX wild-type (top panel) and *W^e^/W^e^* mutant (bottom panel) embryos for *oncRNA6*, *Lrrc34*, *Lypd6* and *Magi2* showed that the expression of *oncRNA6* and *Lrrc34* but not *Lypd6* and *Magi2* is dependent on the presence of germ cells. Scale bar, 100 µm.
(TIF)

Figure S4 **Expression analysis of** ***Dmrtc1c1*** **and** ***Spdya*** **.** Whole-mount ISH (**A**) of XX and XY mouse embryonic gonads from 11.5 to 15.5 dpc and ISH (**B**, **C**) with sagittal sections of XX and XY mouse embryos at 13.5 dpc, as well as section ISH (purple staining) followed by IHC (brown staining) for the germ cell marker E-cadherin of 13.5 dpc ovaries (**C**) demonstrated that *Dmrtc1c1* (**A**, **B**) and *Spdya* (**B**, **C**) are XX germ cell-specifically expressed in an anterior-to-posterior wave from 13.5 dpc. Scale bar, 100 µm (**B**), 50 µm (**C**).
(TIF)

Figure S5 **Expression analysis of somatic cell genes.** ISH with sagittal section of XX and XY mouse embryos at 12.5 dpc showed that *Lypd6*, *Magi2* and *Egfl6* are expressed in ovarian somatic cells. Scale bar, 100 µm.
(TIF)

Figure S6 **Summary of observed expression patterns in the developing ovary.** Schematic representation of the observed expression patterns in the developing ovary, with the expression in ovarian somatic cells marked as grey shading, expression in ovarian germ cells indicted by grey shading within the three circles. Anterior pole is at the top and mesonephric site at the left.
(TIF)

Table S1 **Differentially expressed genes during gonad development.** Excel file containing information for the selected probes (column 1), including the gene name, gene description, EntrezGene Database Identifier, chromosomal localization, biological process, molecular function and cellular component terms from GeneOntology (columns 2 to 8) as well as expression group based on qRT-PCR.
(XLSX)

Table S2 **Differentially expressed protein-coding and non-coding genes as determined by microarray analysis.** Excel file containing information for all differentially expressed protein-coding and non-coding genes identified by microarray analysis using the NCode Mouse array and RNA from mouse embryonic gonads from 11.5 to 14.5 dpc.
(XLSX)

Table S3 **Top ncRNAs with higher expression in the ovary compared to testis at 11.5, 12.5 and 13.5 dpc.** Excel file listing non-coding RNAs that are expressed at higher levels in the ovary compared to testis at 11.5, 12.5 and/or 13.5 dpc identified by microarray analysis using the NCode Mouse array. Non-coding RNA candidates that were validated by qRT-PCR are marked in red. Assignment to expression group is based on cluster analysis and qRT-PCR (in brackets and red).
(XLSX)

Table S4 **Primer sequences used in qRT-PCR**
(DOC)
