# Supplementary figures and images for: Identification of Novel Markers of Mouse Fetal Ovary Development
Source: PLoS One. 2012 Jul 26;7(7):e41683. doi: 10.1371/journal.pone.0041683 (PMC3406020; doi:10.1371/journal.pone.0041683)

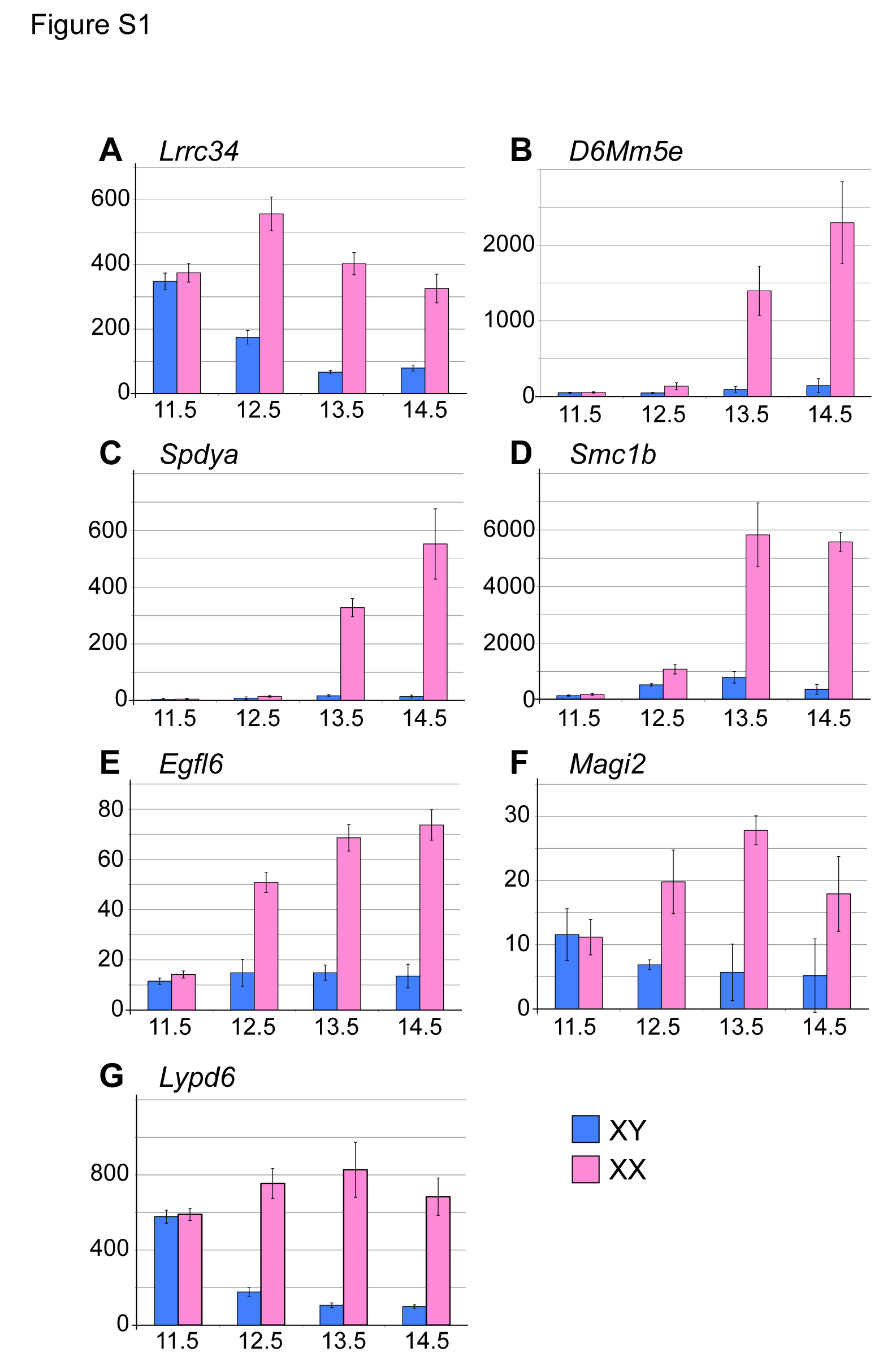

Supplement: Figure S1 — Expression profiles of differentially expressed genes. Normalized microarray expression data (mean ± standard deviation of four independent pools of isolated gonads) of differentially expressed mRNAs, Lrrc34 (A), D6Mm5e (B), Spdya (C), Smc1b (D), Egfl6 (E), Magi2 (F), and Lypd6 (G). (TIF) [file pone.0041683.s001.tif]

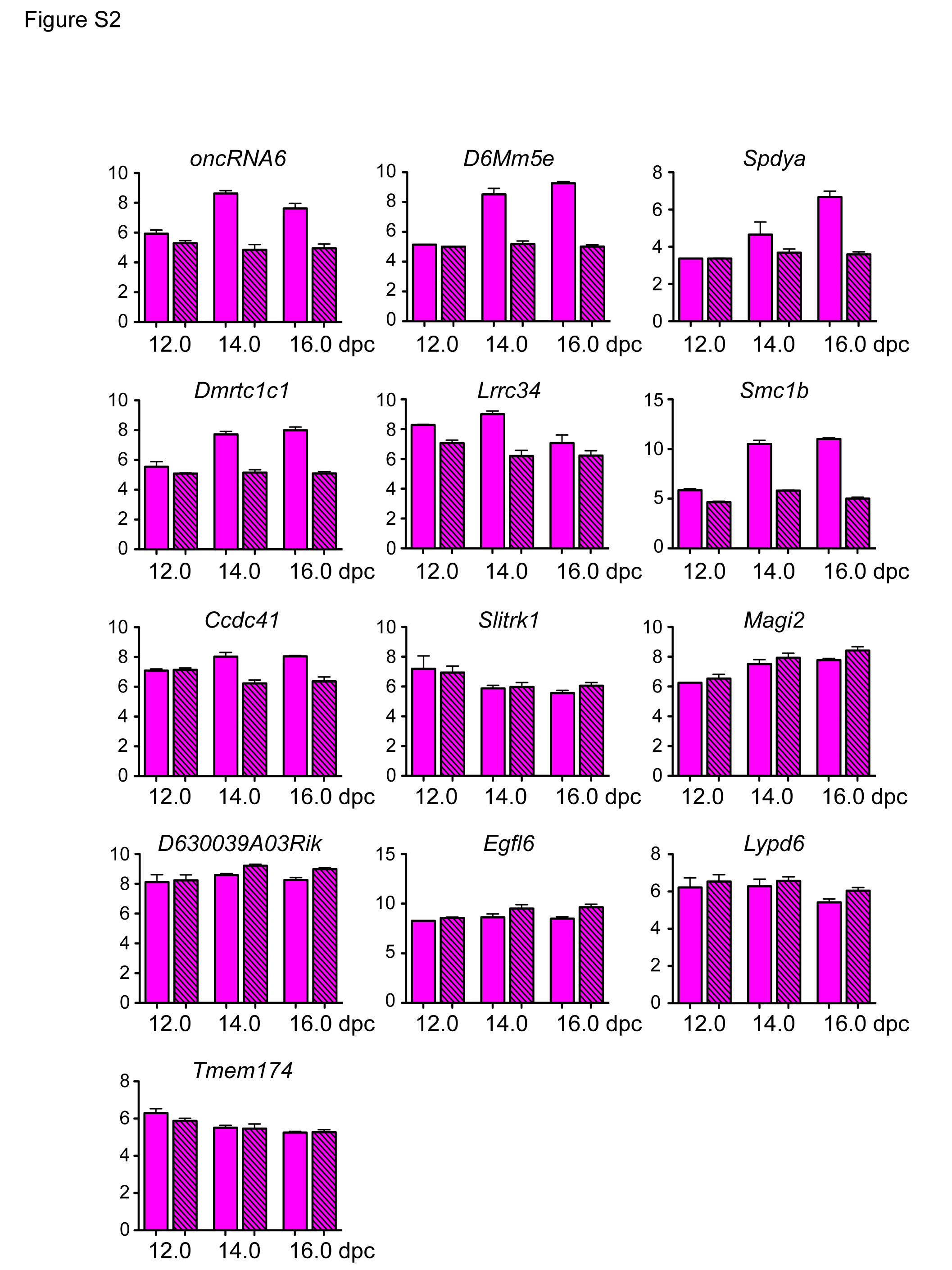

Supplement: Figure S2 — Expression analysis in Wv/Wv mutant mouse ovaries. Microarray analysis of candidate genes comparing expression levels in wild type (solid pink bars) and mutant (cross-hatched pink bars) at 12.0, 14.0 and 16.0 dpc suggested that the expression of oncRNA6, D6Mm5e, Spdya, Dmrtc1c1, Lrrc34, Smc1b and Ccdc41 is dependent on the presence of germ cells. y-axis, relative expression levels on a Log2 scale [36]. (TIF) [file pone.0041683.s002.tif]

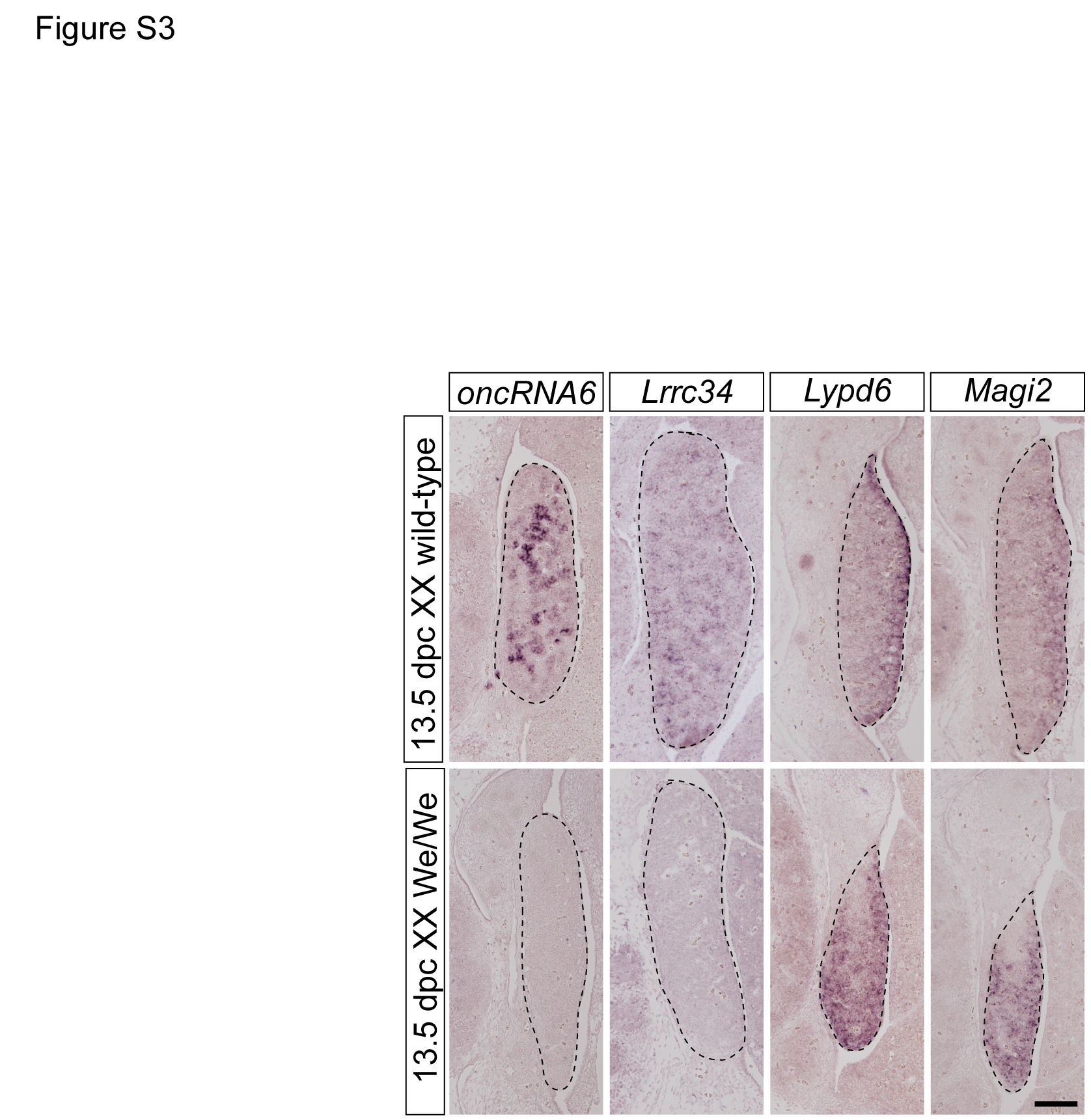

Supplement: Figure S3 — Expression analysis in We/We mutant mouse ovaries. ISH with sagittal section of 13.5 dpc XX wild-type (top panel) and We/We mutant (bottom panel) embryos for oncRNA6, Lrrc34, Lypd6 and Magi2 showed that the expression of oncRNA6 and Lrrc34 but not Lypd6 and Magi2 is dependent on the presence of germ cells. Scale bar, 100 µm. (TIF) [file pone.0041683.s003.tif]

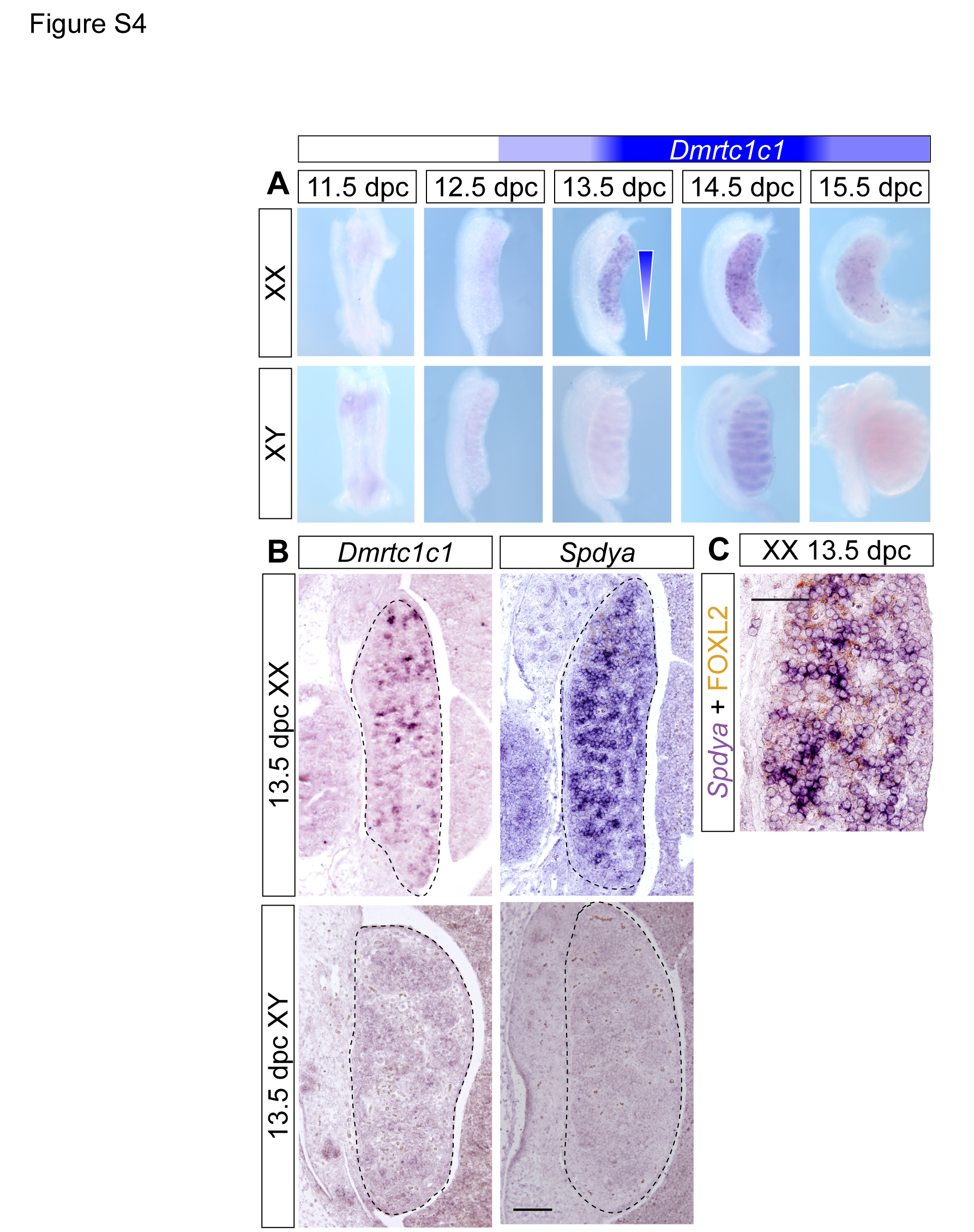

Supplement: Figure S4 — Expression analysis of Dmrtc1c1 and Spdya . Whole-mount ISH (A) of XX and XY mouse embryonic gonads from 11.5 to 15.5 dpc and ISH (B, C) with sagittal sections of XX and XY mouse embryos at 13.5 dpc, as well as section ISH (purple staining) followed by IHC (brown staining) for the germ cell marker E-cadherin of 13.5 dpc ovaries (C) demonstrated that Dmrtc1c1 (A, B) and Spdya (B, C) are XX germ cell-specifically expressed in an anterior-to-posterior wave from 13.5 dpc. Scale bar, 100 µm (B), 50 µm (C). (TIF) [file pone.0041683.s004.tif]

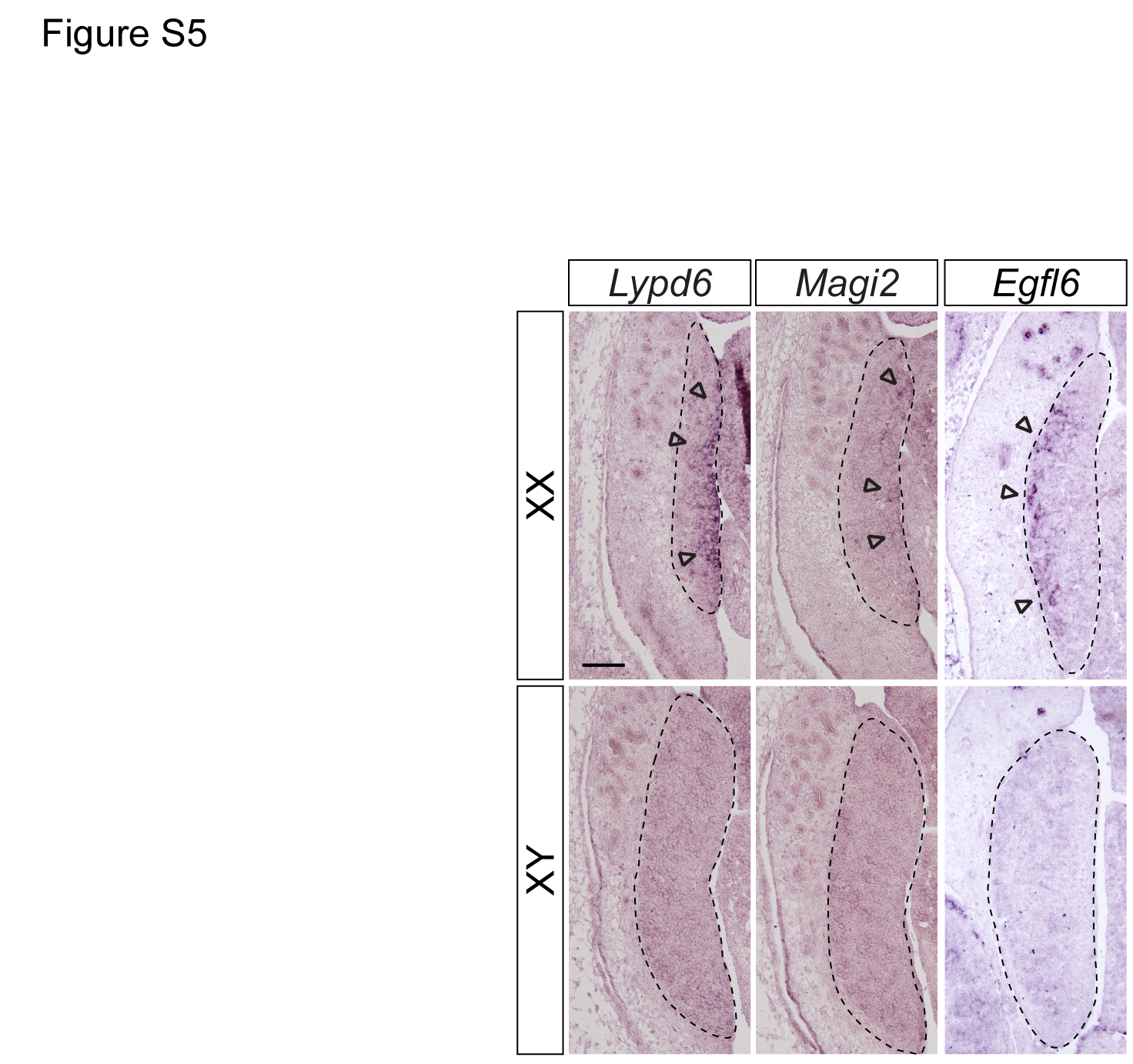

Supplement: Figure S5 — Expression analysis of somatic cell genes. ISH with sagittal section of XX and XY mouse embryos at 12.5 dpc showed that Lypd6, Magi2 and Egfl6 are expressed in ovarian somatic cells. Scale bar, 100 µm. (TIF) [file pone.0041683.s005.tif]

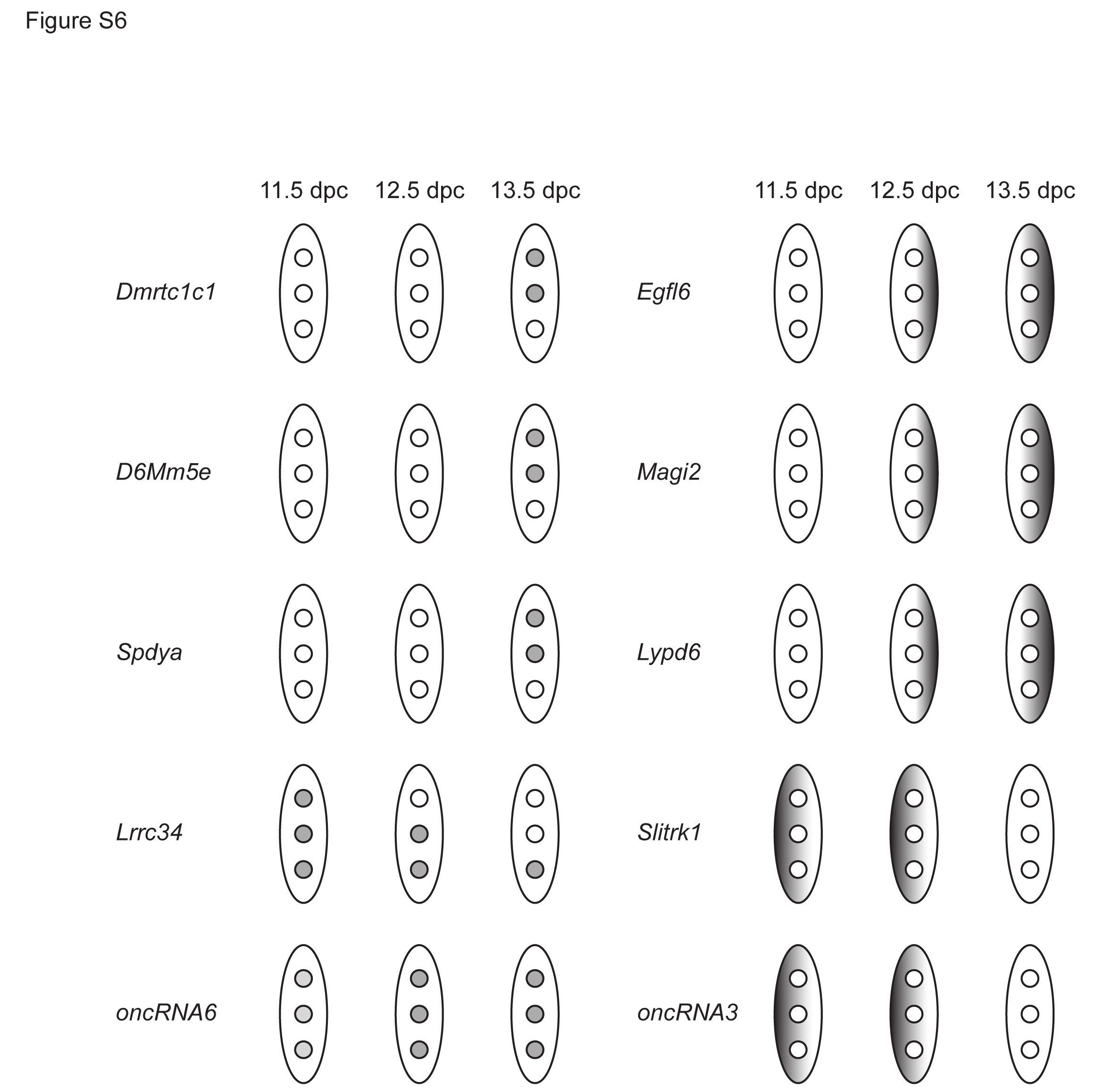

Supplement: Figure S6 — Summary of observed expression patterns in the developing ovary. Schematic representation of the observed expression patterns in the developing ovary, with the expression in ovarian somatic cells marked as grey shading, expression in ovarian germ cells indicted by grey shading within the three circles. Anterior pole is at the top and mesonephric site at the left. (TIF) [file pone.0041683.s006.tif]
