# Supplementary material for: Identification of Novel Markers of Mouse Fetal Ovary Development
Source: PLoS One. 2012 Jul 26;7(7):e41683. doi: 10.1371/journal.pone.0041683 (PMC3406020; doi:10.1371/journal.pone.0041683)
Supplement: Table S4 — Primer sequences used in qRT-PCR (DOC) [file pone.0041683.s010.doc]

**Table S1. Sequences of primers used in qRT-PCR**

| **Gene Symbol** | **Primer Name** | **Primer Sequence (5’-3’)** |
| --- | --- | --- |
| *Spdya* | SpdyaRT_F | gcctgaggtagatcgctgag |
|  | SpdyaRT_R | cgcattttagcttcccaaga |
| *Adam8* | Adam8RT_F | ctccagatcccatcatgctt |
|  | Adam8RT_R | ggtccaggggctactgct |
| *Lrrc34* | Lrrc34RT_F | gggctctaggcgaaggaa |
|  | Lrrc34RT_R | agcttctccatcgactctgg |
| *D6Mm5e* | D6MmRT_F | acctgaagacagcctaaagatca |
|  | D6MmRT_R | gcagagggttgtaggtgagc |
| *Slitrk1* | SlitrRT_F | ttcctccctcagacaactcg |
|  | SlitrRT_R | ctgctctttcggaattacgtg |
| *Dmrtc1c1* | DmrtcRT_F | gggcttttgctgagtcca |
|  | DmrtcRT_R | gtggtgatcggtggaggta |
| *AK015184* | 2215RT_F | tgaatgctggcacactacttg |
|  | 2215RT_R | ccccaagtcatccacatagc |
| *AK019493* | 19493RT_F | ggagaagggagagccacaat |
|  | 19493RT_R | ataaatgagtgacagcgaatgg |
| *Egfl6* | RT_Egf_F | tggggtctgtcagtatggaac |
|  | RT_Egf_R | cctgggctcacacatagctt |
| *Ccdc41* | qPCR_Ccdc41_F | gaaaatgaaatacaagtcagaggttg |
|  | qPCR_Ccdc41_R | ttcgtttgctgtctctcgtg |
| *D630039A03Rik* | qPCR_D63_F | cctgtaaacatccagtttcttcg |
|  | qPCR_D63_R | gagtaggcatggtcccttctt |
| *Fam196b* | qPCR_Fam196b_F | gaacacagcatgcatcatatacagt |
|  | qPCR_Fam196b_R | gcttcctcagttggttctgc |
| *Lypd6* | qPCR_Lypd6_F | ctacctccacccttcaacca |
|  | qPCR_Lypd6_R | gatgtcaggagcccatcg |
| *Magi2* | qPCR_Magi2_F | gggaaaatggagacaggtgat |
|  | qPCR_Magi2_R | tgaccaataggaacagactgga |
| *Smc1b* | qPCR_Smc1b_F | tgctggaattgataaccatgag |
|  | qPCR_Smc1b_R | agatcaagcagtcttccaaacac |
| *Tmem174* | qPCR_Tmem174_F | agtccccctcagtactacacca |
|  | qPCR_Tmem174_R | catcagagtcgggcctgt |
| *Sdha* | qPCR_Sdha_F | tgttcagttccaccccaca |
|  | qPCR_Sdha_R | tctccacgacacccttctgt |
| *AK046039* | AK046039_qPCR_f | aaaggcattgtgtgtgtgtca |
|  | AK046039_qPCR_r | tgcaaaccagcatttttatcc |

| *AK015693* | AKjp21_F | ataggttcgccaccattcag |
| --- | --- | --- |
|  | AKjp21_R | gttgccgttttcaggtgact |
| *AK036014* | AK036014_qPCR_f | cctcagctcccagctcttg |
|  | AK036014_qPCR_r | aattctgcatgtccacaaagg |
| *AK017289* | AKjp10a_F | gctccagatgggtgtgtttt |
|  | AKjp10a_R | acaacggggtctacagtgct |
| *AK034059* | AK034059_qPCR_f | ttgactgcactgggtcagag |
|  | AK034059_qPCR_r | tgggctttgtagacacatgg |
| *AK015136* | AK015136_qPCR_f | tgaccacaaaacatcatattcaca |
|  | AK015136_qPCR_r | ttccaggctagaactcaactcc |
| *AK014986* | AK014986_qPCR_f | gtggagctggcctgaatc |
|  | AK014986_qPCR_r | cgagctgtagtgatcccagtc |
| *AK021294* | AKjp19_F | ggtttggtgaaaacgcaaag |
|  | AKjp19_R | attttcgcgcaatgagaact |
| *AK156088* | AK156088_qPCR_f | tcattttcatccctgttgacc |
|  | AK156088_qPCR_r | ggaatcccacagtcaccatc |
